# Supplementary material for: Pyruvate Dehydrogenase Kinase 1 inhibition mediated oxidative phosphorylation enhancement in cartilage promotes osteoarthritis progression
Source: BMC Musculoskelet Disord. 2023 Jul 20;24:597. doi: 10.1186/s12891-023-06585-6 (PMC10357736; doi:10.1186/s12891-023-06585-6)
Supplement: Supplementary file 1 — Additional file 1. [file 12891_2023_6585_MOESM1_ESM.zip › BP.Gsea.1653623667859/GOBP_NUCLEOTIDE_EXCISION_REPAIR.html]

Details for gene set GOBP\_NUCLEOTIDE\_EXCISION\_REPAIR[GSEA]

|  || Dataset | 55457\_collapsed\_to\_symbols.cls.cls #OA\_versus\_normal.cls.cls #OA\_versus\_normal\_repos |
| Phenotype | cls.cls#OA\_versus\_normal\_repos |
| Upregulated in class | OA |
| GeneSet | GOBP\_NUCLEOTIDE\_EXCISION\_REPAIR |
| Enrichment Score (ES) | 0.36148697 |
| Normalized Enrichment Score (NES) | 1.4858242 |
| Nominal p-value | 0.033591732 |
| FDR q-value | 0.39038324 |
| FWER p-Value | 1.0 |
Table: GSEA Results Summary

  

Fig 1: Enrichment plot: GOBP\_NUCLEOTIDE\_EXCISION\_REPAIR      
 Profile of the Running ES Score & Positions of GeneSet Members on the Rank Ordered List

  

| SYMBOL | TITLE | RANK IN GENE LIST | RANK METRIC SCORE | RUNNING ES | CORE ENRICHMENT || 1 | RPA3 | replication protein A3 [Source:HGNC Symbol;Acc:HGNC:10291] | 13 | 1.159 | 0.0575 | Yes |
| 2 | POLB | DNA polymerase beta [Source:HGNC Symbol;Acc:HGNC:9174] | 93 | 0.923 | 0.0980 | Yes |
| 3 | CETN2 | centrin 2 [Source:HGNC Symbol;Acc:HGNC:1867] | 130 | 0.875 | 0.1393 | Yes |
| 4 | RPA2 | replication protein A2 [Source:HGNC Symbol;Acc:HGNC:10290] | 339 | 0.727 | 0.1598 | Yes |
| 5 | SLC30A9 | solute carrier family 30 member 9 [Source:HGNC Symbol;Acc:HGNC:1329] | 390 | 0.710 | 0.1918 | Yes |
| 6 | OGG1 | 8-oxoguanine DNA glycosylase [Source:HGNC Symbol;Acc:HGNC:8125] | 453 | 0.681 | 0.2213 | Yes |
| 7 | ERCC8 | "ERCC excision repair 8, CSA ubiquitin ligase complex subunit [Source:HGNC Symbol;Acc:HGNC:3439]" | 506 | 0.656 | 0.2504 | Yes |
| 8 | RPA1 | replication protein A1 [Source:HGNC Symbol;Acc:HGNC:10289] | 571 | 0.632 | 0.2773 | Yes |
| 9 | POLR2I | RNA polymerase II subunit I [Source:HGNC Symbol;Acc:HGNC:9196] | 640 | 0.609 | 0.3027 | Yes |
| 10 | POLD1 | "DNA polymerase delta 1, catalytic subunit [Source:HGNC Symbol;Acc:HGNC:9175]" | 811 | 0.566 | 0.3181 | Yes |
| 11 | GTF2H5 | general transcription factor IIH subunit 5 [Source:HGNC Symbol;Acc:HGNC:21157] | 1032 | 0.511 | 0.3267 | Yes |
| 12 | KAT7 | lysine acetyltransferase 7 [Source:HGNC Symbol;Acc:HGNC:17016] | 1125 | 0.491 | 0.3444 | Yes |
| 13 | XPA | "XPA, DNA damage recognition and repair factor [Source:HGNC Symbol;Acc:HGNC:12814]" | 1615 | 0.411 | 0.3271 | Yes |
| 14 | PNKP | polynucleotide kinase 3'-phosphatase [Source:HGNC Symbol;Acc:HGNC:9154] | 1662 | 0.404 | 0.3439 | Yes |
| 15 | ERCC4 | "ERCC excision repair 4, endonuclease catalytic subunit [Source:HGNC Symbol;Acc:HGNC:3436]" | 1696 | 0.400 | 0.3615 | Yes |
| 16 | POLA1 | "DNA polymerase alpha 1, catalytic subunit [Source:HGNC Symbol;Acc:HGNC:9173]" | 2300 | 0.327 | 0.3310 | No |
| 17 | DDB1 | damage specific DNA binding protein 1 [Source:HGNC Symbol;Acc:HGNC:2717] | 2302 | 0.326 | 0.3474 | No |
| 18 | LIG4 | DNA ligase 4 [Source:HGNC Symbol;Acc:HGNC:6601] | 2668 | 0.283 | 0.3333 | No |
| 19 | ERCC1 | "ERCC excision repair 1, endonuclease non-catalytic subunit [Source:HGNC Symbol;Acc:HGNC:3433]" | 2948 | 0.255 | 0.3245 | No |
| 20 | NTHL1 | nth like DNA glycosylase 1 [Source:HGNC Symbol;Acc:HGNC:8028] | 3219 | 0.228 | 0.3150 | No |
| 21 | DDB2 | damage specific DNA binding protein 2 [Source:HGNC Symbol;Acc:HGNC:2718] | 3257 | 0.225 | 0.3235 | No |
| 22 | XPC | "XPC complex subunit, DNA damage recognition and repair factor [Source:HGNC Symbol;Acc:HGNC:12816]" | 3278 | 0.222 | 0.3332 | No |
| 23 | ERCC2 | "ERCC excision repair 2, TFIIH core complex helicase subunit [Source:HGNC Symbol;Acc:HGNC:3434]" | 3544 | 0.198 | 0.3226 | No |
| 24 | BRCA2 | BRCA2 DNA repair associated [Source:HGNC Symbol;Acc:HGNC:1101] | 3853 | 0.170 | 0.3072 | No |
| 25 | ERCC5 | "ERCC excision repair 5, endonuclease [Source:HGNC Symbol;Acc:HGNC:3437]" | 4380 | 0.122 | 0.2724 | No |
| 26 | MNAT1 | MNAT1 component of CDK activating kinase [Source:HGNC Symbol;Acc:HGNC:7181] | 4681 | 0.098 | 0.2540 | No |
| 27 | FANCC | FA complementation group C [Source:HGNC Symbol;Acc:HGNC:3584] | 5138 | 0.061 | 0.2216 | No |
| 28 | TP53 | tumor protein p53 [Source:HGNC Symbol;Acc:HGNC:11998] | 5511 | 0.032 | 0.1942 | No |
| 29 | RNF111 | ring finger protein 111 [Source:HGNC Symbol;Acc:HGNC:17384] | 5711 | 0.015 | 0.1795 | No |
| 30 | BRIP1 | BRCA1 interacting helicase 1 [Source:HGNC Symbol;Acc:HGNC:20473] | 5885 | 0.003 | 0.1661 | No |
| 31 | POLL | DNA polymerase lambda [Source:HGNC Symbol;Acc:HGNC:9184] | 6214 | -0.022 | 0.1417 | No |
| 32 | GTF2H4 | general transcription factor IIH subunit 4 [Source:HGNC Symbol;Acc:HGNC:4658] | 6295 | -0.027 | 0.1368 | No |
| 33 | FAN1 | FANCD2 and FANCI associated nuclease 1 [Source:HGNC Symbol;Acc:HGNC:29170] | 6323 | -0.030 | 0.1362 | No |
| 34 | CUL4A | cullin 4A [Source:HGNC Symbol;Acc:HGNC:2554] | 6701 | -0.055 | 0.1097 | No |
| 35 | MMS19 | "MMS19 homolog, cytosolic iron-sulfur assembly component [Source:HGNC Symbol;Acc:HGNC:13824]" | 6733 | -0.058 | 0.1102 | No |
| 36 | HUS1 | HUS1 checkpoint clamp component [Source:HGNC Symbol;Acc:HGNC:5309] | 7340 | -0.105 | 0.0684 | No |
| 37 | POLD3 | "DNA polymerase delta 3, accessory subunit [Source:HGNC Symbol;Acc:HGNC:20932]" | 7568 | -0.125 | 0.0570 | No |
| 38 | ERCC6 | "ERCC excision repair 6, chromatin remodeling factor [Source:HGNC Symbol;Acc:HGNC:3438]" | 7939 | -0.150 | 0.0358 | No |
| 39 | ATXN3 | ataxin 3 [Source:HGNC Symbol;Acc:HGNC:7106] | 8452 | -0.190 | 0.0055 | No |
| 40 | ERCC3 | "ERCC excision repair 3, TFIIH core complex helicase subunit [Source:HGNC Symbol;Acc:HGNC:3435]" | 8669 | -0.206 | -0.0009 | No |
| 41 | RAD23B | "RAD23 homolog B, nucleotide excision repair protein [Source:HGNC Symbol;Acc:HGNC:9813]" | 8777 | -0.215 | 0.0016 | No |
| 42 | XAB2 | XPA binding protein 2 [Source:HGNC Symbol;Acc:HGNC:14089] | 8778 | -0.215 | 0.0124 | No |
| 43 | SIRT1 | sirtuin 1 [Source:HGNC Symbol;Acc:HGNC:14929] | 8947 | -0.227 | 0.0108 | No |
| 44 | RAD23A | "RAD23 homolog A, nucleotide excision repair protein [Source:HGNC Symbol;Acc:HGNC:9812]" | 9028 | -0.234 | 0.0164 | No |
| 45 | GTF2H1 | general transcription factor IIH subunit 1 [Source:HGNC Symbol;Acc:HGNC:4655] | 9455 | -0.269 | -0.0031 | No |
| 46 | POLE | "DNA polymerase epsilon, catalytic subunit [Source:HGNC Symbol;Acc:HGNC:9177]" | 11026 | -0.433 | -0.1035 | No |
| 47 | GTF2H3 | general transcription factor IIH subunit 3 [Source:HGNC Symbol;Acc:HGNC:4657] | 12001 | -0.593 | -0.1494 | No |
| 48 | CETN1 | centrin 1 [Source:HGNC Symbol;Acc:HGNC:1866] | 12015 | -0.596 | -0.1203 | No |
| 49 | RAD52 | "RAD52 homolog, DNA repair protein [Source:HGNC Symbol;Acc:HGNC:9824]" | 12134 | -0.627 | -0.0978 | No |
| 50 | USP7 | ubiquitin specific peptidase 7 [Source:HGNC Symbol;Acc:HGNC:12630] | 12289 | -0.673 | -0.0758 | No |
| 51 | RPA4 | replication protein A4 [Source:HGNC Symbol;Acc:HGNC:30305] | 12406 | -0.709 | -0.0490 | No |
| 52 | GTF2H2 | general transcription factor IIH subunit 2 [Source:HGNC Symbol;Acc:HGNC:4656] | 12612 | -0.806 | -0.0243 | No |
| 53 | GTF2H2C | GTF2H2 family member C [Source:HGNC Symbol;Acc:HGNC:31394] | 12765 | -0.924 | 0.0105 | No |
Table: GSEA details [plain text format]

  

Fig 2: GOBP\_NUCLEOTIDE\_EXCISION\_REPAIR      
 Blue-Pink O' Gram in the Space of the Analyzed GeneSet

  

Fig 3: GOBP\_NUCLEOTIDE\_EXCISION\_REPAIR: Random ES distribution      
 Gene set null distribution of ES for **GOBP\_NUCLEOTIDE\_EXCISION\_REPAIR**
